# Interpretable Machine Learning for One-Part Fly-Ash/Slag Geopolymer Strength Prediction: Toward Multifunctional Binder Design

**DOI:** 10.3390/ma19153347

**Published:** 2026-08-06

**Authors:** Vinoth Nageshwaran, Sudhir Amritphale, Soundararajan Ezekiel

**Affiliations:** 1 School of Computer and Information Sciences, University of the Cumberlands, 6178 College Station Drive, Williamsburg, KY 40769, USA; 2 Science Investments LLC, Ruston, LA 71270, USA; amritphale57@gmail.com; 3 Department of Mathematical and Computer Sciences, Indiana University of Pennsylvania, Indiana, PA 15705, USA; sezekiel@iup.edu

**Keywords:** one-part geopolymer, machine learning, alkali-activated materials, multifunctional composites, mix design optimization, defence infrastructure

## Abstract

Portland cement production accounts for roughly 8% of anthropogenic CO_2_ emissions, driving interest in low-carbon geopolymer binders. One-part (“just-add-water”) geopolymers, which replace hazardous liquid activators with a dry, pre-blended solid activator, are especially suited to field deployment where handling safety and logistics are decisive. However, their formulation space is combinatorially vast, and trial-and-error development cannot efficiently navigate it. This paper reviews one-part geopolymer science, presents a new comparative and interpretable ML analysis of a published 80-mixture one-part fly-ash/ground granulated blast-furnace slag (GGBS, hereafter slag) geopolymer dataset from twelve studies, and proposes an AI-assisted design framework. The ML demonstration targets 28-day compressive strength only. Under leave-one-source-out (LOSO) cross-validation—the appropriate test for a literature-pooled dataset—gradient-boosted trees achieved R^2^ = 0.61 (RMSE = 15.5 MPa; 95% bootstrap confidence interval on R^2^, 0.44–0.75), well above a linear baseline (0.36), suggesting that non-linear structure transfers across studies; a random split gives a higher but less reliable R^2^ = 0.90 on only 16 test mixtures. Because fly-ash and slag contents are near-perfectly anti-correlated (r=−0.99), we model the precursor axis as a single slag fraction descriptor; SHAP then identifies this precursor balance and the activator’s Na_2_O dosage as the dominant statistical predictors of strength in this dataset, an ordering consistent with known activation chemistry; causal confirmation of these associations awaits the experimental validation stage of the proposed framework. Demonstrated for strength only, at paste level, the framework offers a transferable route toward multifunctional low-carbon binders for protective and infrastructure applications; the multifunctional extensions are proposed, but not yet demonstrated.

## 1. Introduction

Concrete is the most-consumed manufactured material on Earth, and the ordinary Portland cement (OPC) that binds it is responsible for approximately 8% of global anthropogenic carbon dioxide emissions—a share arising from both the calcination of limestone and the fossil energy required to reach clinkering temperatures. As national decarbonization commitments tighten and as the construction sector confronts the scale of its embodied-carbon footprint, the search for binders that deliver OPC-comparable mechanical performance at a fraction of the emissions has become one of the defining materials challenges of the coming decades. Alkali-activated materials (AAMs) and geopolymers—synthesized by reacting aluminosilicate precursors such as fly ash, ground granulated blast-furnace slag (GGBS; hereafter, “slag” and “GGBS” are used interchangeably for this material), or metakaolin with an alkaline activator—are the most technologically mature low-carbon binder class, capable of 40–80% reductions in cradle-to-gate CO_2_ relative to OPC while reusing industrial by-products that would otherwise be landfilled; fly ash is likewise increasingly valorized in adjacent low-carbon material streams such as cemented coal-gangue/fly-ash mine backfill, where aggregate gradation and pore structure govern mechanical performance [1].

Despite three decades of research, conventional “two-part” geopolymers face a persistent barrier to field adoption: they require a concentrated liquid alkaline activator, typically a sodium or potassium hydroxide/silicate solution. These solutions are corrosive, viscous, exothermic on preparation, and hazardous to handle, and they cannot be dry-bagged, shipped, and stored like OPC. This constraint is disqualifying precisely in the settings where a rapidly deployable, low-carbon, high-performance binder would be most valuable—forward operating bases, disaster-response reconstruction, remote infrastructure, and protective structures—where trained personnel, controlled mixing environments, and hazardous-material logistics are unavailable. The activator, in short, is the bottleneck standing between geopolymer science and field practice.

One-part, or “just-add-water,” geopolymers dissolve this bottleneck. By pre-blending a solid activator—commonly anhydrous sodium metasilicate, sodium aluminate, or a calcined/mechanochemically activated alkali source—with the aluminosilicate precursor into a single dry powder, the one-part approach reproduces the OPC use-model exactly: the end user adds only water. This transfers all hazardous chemistry upstream into a controlled manufacturing facility and yields a shelf-stable, transportable binder amenable to bagging, spraying, and even 3D printing in the field. Since the foundational demonstrations of ambient-cured one-part mixes in the mid-2010s [2,3], the sub-field has grown rapidly, with formulations now spanning fly-ash, slag, red mud, and natural pozzolan precursors [4,5] and functional variants including fibre-reinforced, engineered-cementitious, foamed, and printable composites [6,7,8].

Yet this very versatility creates a design problem. The performance of a one-part geopolymer is governed by a high-dimensional, strongly interacting formulation space: precursor identity and blend ratio, solid activator type, dosage, and silica modulus, water-to-binder ratio, functional additives (fibres, fillers, conductive or shielding phases), and curing regime. These variables interact non-linearly—the optimal activator dosage depends on precursor reactivity; the benefit of heat curing depends on the fly-ash/slag balance; workability, strength, and durability trade against one another. The combinatorial size of this space defeats the one-factor-at-a-time experimentation that still dominates the literature, and it explains why property optimization in one-part systems remains slow, sample-hungry, and poorly transferable between laboratories.

Machine learning (ML) offers a direct response to this challenge. Over the past five years, data-driven models—artificial neural networks, tree ensembles, support vector machines, and gene expression programming—have been applied with growing success to predict the compressive strength and, less often, the durability and fresh-state properties of alkali-activated concretes [9,10,11,12], and interpretability methods such as SHAP (SHapley Additive exPlanations) are beginning to convert these models from black-box predictors into sources of design insight [13,14]. A bibliometric survey we conducted for this work (a title-level trend scan rather than a systematic review; 1850 geopolymer papers, 2014–mid-2026; Section 1.1) confirms both the momentum and the gap: ML-assisted and one-part research are the two fastest-growing streams in the field, yet their explicit intersection remains a small, if rapidly growing, cluster of roughly two dozen papers concentrated in the last three years, and a concept co-occurrence analysis over paper titles shows the strongest ML associations running to compressive strength prediction of conventional two-part mixes, with markedly weaker title-level coupling to one-part formulation and essentially none to fibre-reinforcement or thermal/functional performance. At the title level, the literature map suggests that machine learning has only recently begun to be applied explicitly to the one-part design problem, and scarcely at all to the multifunctional properties that defence and infrastructure applications demand.

This paper addresses that gap on three fronts. First, we review the materials science of one-part geopolymer composites—precursor and activator chemistry, reaction mechanisms, mechanical and durability behaviour, and the emerging multifunctional variants relevant to protective and civil infrastructure. Second, we present a new comparative and interpretable ML analysis of a published 80-mixture one-part fly-ash/GGBS geopolymer dataset (compiled from twelve independent studies): the dataset is not new, but the comparative modelling, leave-one-source-out evaluation, and SHAP interpretation are. We train and compare four regression algorithms, evaluate them by both random split and leave-one-source-out cross-validation, and apply SHAP analysis to extract design-actionable feature effects for the compressive strength target. Third, we synthesize these threads into a generalizable, interpretable AI-assisted design framework that links mixture descriptors to multifunctional performance targets and closes the loop from data curation through model interpretation to experimental validation. Our aim is not a single optimized mix but a transferable methodology for the rational, accelerated design of the field-deployable low-carbon binders that defence and civil infrastructure increasingly require.

### 1.1. Bibliometric Methodology

**Figure 1:**
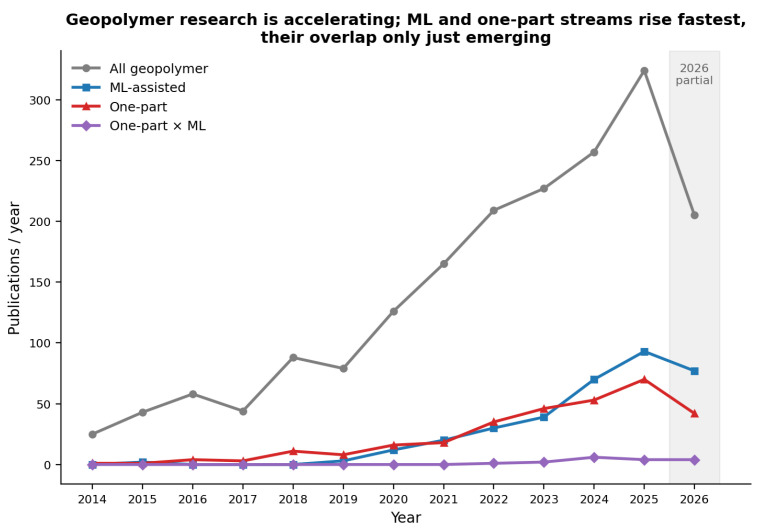
Publication trends in the geopolymer field by sub-stream (2014–mid-2026, n=1850; 2026 is a partial year, shaded). Geopolymer research is accelerating overall, with the ML-assisted and one-part streams the fastest-growing; their explicit intersection (one-part × ML) remains a small but only recently emerging cluster.

**Figure 2:**
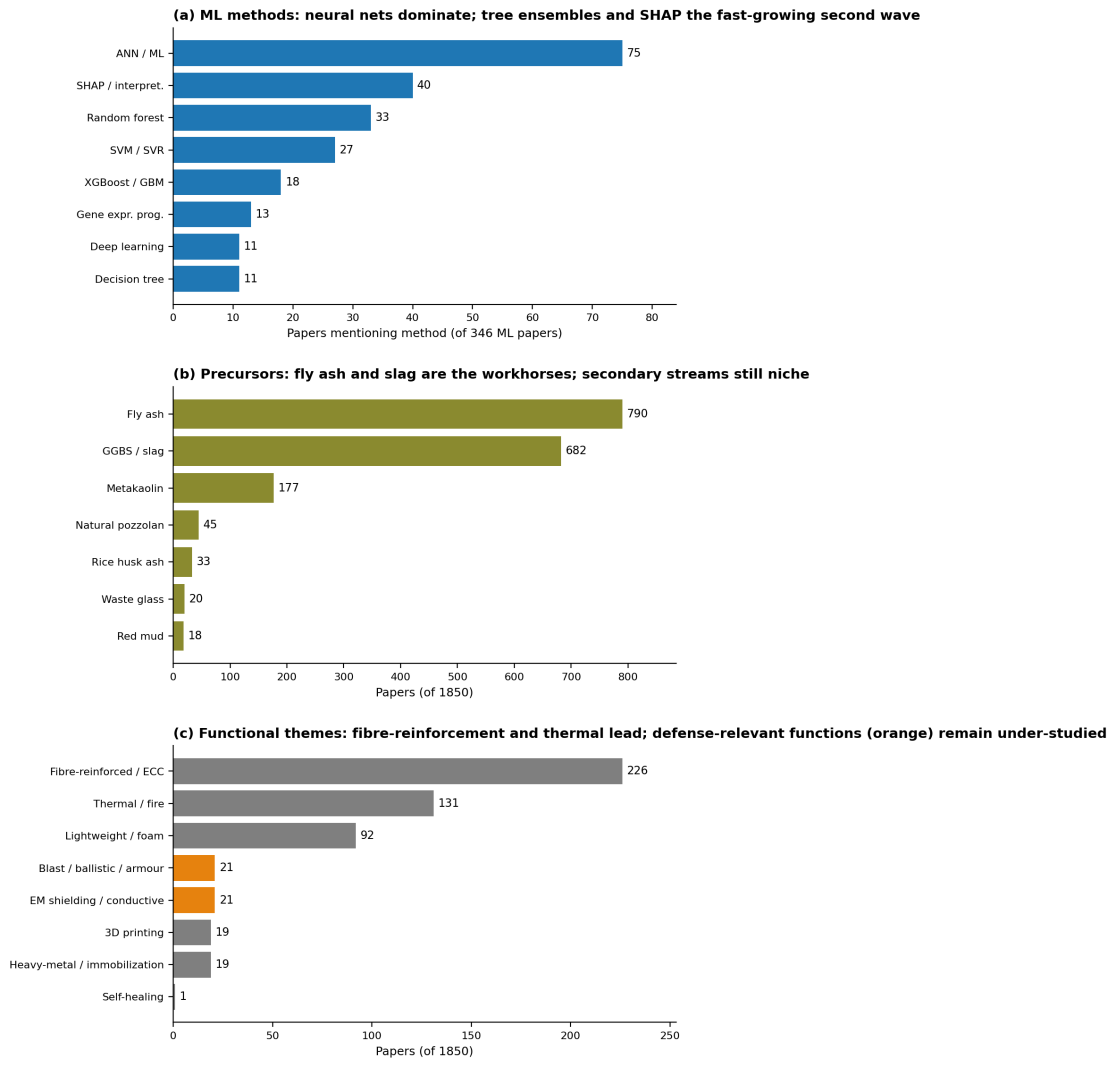
Thematic composition of the geopolymer corpus. (**a**) ML methods mentioned across the 346 ML-tagged papers; (**b**) precursors and (**c**) functional themes across all 1850 on-topic papers. Neural networks dominate the methods and fly ash and slag the precursors, while defence-relevant functional themes (orange) remain under-studied.

**Figure 3:**
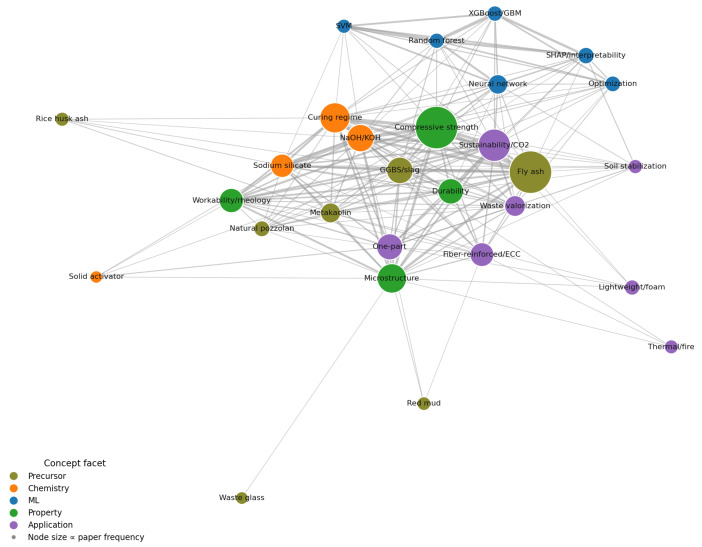
Concept co-occurrence network of the corpus (1092 papers with title text longer than 120 characters). Machine learning methods (blue) cluster together and couple to the mix design/strength core, but not to one-part formulation or multifunctional properties.

The literature map in Figure 1, Figure 2 and Figure 3 was built from a reproducible query of the Crossref REST API, retrieved on 1 July 2026, filtered to from-pub-date:2014-01-01,type:journal-article, with cursor pagination (200 records per page). Eight query.bibliographic strings were issued: (1) “geopolymer machine learning”; (2) “one-part geopolymer”; (3) “one-part geopolymer compressive strength”; (4) “geopolymer concrete machine learning prediction”; (5) “alkali activated material mix design optimization machine learning”; (6) “geopolymer electromagnetic shielding OR fibrereinforced multifunctional”; (7) “one-part alkali activated slag fly ash”; and (8) “geopolymer artificial neural network compressive strength”. Records were pooled and de-duplicated by DOI, yielding 4312 unique articles.

Relevance and sub-stream tagging used case-insensitive regular expressions applied to the lower-cased, HTML-stripped title text of each record (the corpus retains title-level bibliographic metadata; abstracts were not stored). A record was retained as on-topic if it matched geopolymer|alkali[- ]activat|geopolymeric; that is, the 1850 on-topic papers are the subset of the 4312 de-duplicated records whose titles pass this on-topic regex filter. The ≈2460 records removed at this step are articles returned by the broad bibliographic queries (e.g., general machine learning, concrete, or materials papers) whose titles do not concern geopolymers or alkali activation; no additional screening criteria were applied between de-duplication and relevance tagging. The retained pool spans 2014 to mid-2026. The ML stream matched machine learning|deep learning|neural network|random forest| gradient boost|xgboost|artificial intelligence|ann|svm|gep|ensemble model| predictive model|data[- ]driven|gene expression programming; the one-part stream matched one[- ]part|just[- ]add[- ]water|solid activator|powder activat; a genuine defence-function count required blast|ballistic|armour|penetrat|projectile| military|protective structure|spall|anti[- ]blast while explicitly excluding blast [- ]furnace (which denotes slag, not blast resistance). The full regex vocabulary for the precursor and functional facets accompanies the manuscript as analysis code.

Partial-year handling: 2026 records (retrieval mid-year) are included but flagged as a partial year in the trend panel of Figure 1 and are never annualized or extrapolated. The concept co-occurrence network (Figure 3) was computed over the 1092 records whose title text exceeds 120 characters, using a controlled vocabulary of 33 concepts across five facets; edges are Jaccard co-occurrence weights and communities were detected by the Louvain method (resolution 1.0, seed 42). To gauge tagging quality, we adjudicated a random sample of 100 records independently of the regex tags and compared the two labellings: the regex tags achieved precision = 1.00 for both the ML and one-part streams (no false positives) with recall = 0.76 (ML) and 0.86 (one-part), the residual errors being recall misses on records whose titles omit the trigger terms. Because the retained corpus stores titles rather than full abstracts, this validation is title-based and therefore conservative on recall. A PRISMA-style screening summary (Table S1) and the 100-record validation set accompany the manuscript. Because the corpus derives from title-level text, the absence of an edge indicates the absence of co-mention in titles, not necessarily the absence of any study; the counts map where the field’s attention is concentrated rather than furnishing an exhaustive census. We therefore present the bibliometric component explicitly as a title-level trend scan, not a systematic review: the adjudicated recall values (0.76 for the ML stream, 0.86 for one-part) bound the expected undercount of each stream; abstract-level tagging was not performed because Crossref abstract coverage is partial and abstracts were not retained in the stored corpus, so re-tagging on abstracts is left to future work on a corpus built for that purpose; and the co-occurrence network—restricted by construction to the 1092 records (59%) whose titles are long enough to carry multiple concepts, without correction for title length—should be read as a map of title-level attention, not a census of the literature. None of the manuscript’s quantitative claims about the ML demonstration depend on the bibliometric counts; they motivate the gap statement only.

## 2. One-Part Geopolymer Composites: Materials and Mechanisms

### 2.1. From Two-Part to One-Part Activation

In conventional geopolymerization, dissolution of the aluminosilicate precursor by a concentrated alkaline solution liberates silicate and aluminate monomers that polycondense into an amorphous sodium (or potassium) aluminosilicate hydrate (N-A-S-H) gel; in calcium-rich systems such as slag, a coexisting calcium aluminosilicate hydrate (C-A-S-H) gel forms and typically dominates ambient-temperature strength development. The one-part route preserves this chemistry but delivers the alkalinity from a solid source that dissolves on contact with mixing water. Anhydrous sodium metasilicate is the most widely used solid activator because it supplies both the alkali and the soluble silica needed to tune the silica modulus (SiO_2_/Na_2_O), the single most influential activator parameter [15]; sodium aluminate, potassium carbonate, calcined layered double hydroxides, and mechanochemically or thermally activated blends of the precursor with solid NaOH are also documented [16,17,18]. The engineering consequence is that the reactivity ceiling of a one-part system is generally lower than that of an optimally activated two-part system, and much of the sub-field’s research effort is devoted to recovering that performance gap through precursor selection, activator design, and curing.

### 2.2. Precursors and the Fly-Ash/Slag Axis

The bibliometric evidence is unambiguous about which raw materials the field is built on: fly ash and GGBS together account for the overwhelming majority of published one-part formulations, with metakaolin, red mud, rice-husk ash, natural pozzolans, and waste glass forming a secondary tier. This concentration reflects availability, cost, and a favourable balance of reactive silica and alumina [5,19]. The fly-ash/slag ratio is the primary composition lever: slag-rich mixes cure and gain strength at ambient temperature through C-A-S-H formation but can suffer rapid setting and higher shrinkage, whereas fly-ash-rich mixes are more workable and dimensionally stable but often demand elevated temperature curing to develop strength. Blending the two is the standard route to ambient-cured, workable, high-strength one-part binders, and the fly-ash/slag balance is widely reported as a primary composition-side control on compressive strength—though, as the ML analysis below shows, its measured influence in any given dataset depends on how widely composition is varied relative to curing and mix design factors.

### 2.3. Multifunctional Variants for Defence and Infrastructure

Beyond structural strength, one-part geopolymers are being engineered for functional performance directly relevant to protective and civil infrastructure. Fibre-reinforced and engineered-cementitious one-part composites, incorporating polyethylene, PVA, basalt, or steel fibres, provide the tensile ductility and strain-hardening required for blast- and impact-resistant elements [6,8]. The inherently high thermal stability of the aluminosilicate network makes geopolymers strong candidates for fire- and heat-resistant linings—a pertinent advantage given that ordinary concrete suffers severe, microstructurally driven strength deterioration at elevated temperature (in situ characterization reports compressive strength losses of roughly 28–81% between 400 and 800 °C as hydration products decompose and microcracks propagate [20]). Foamed and lightweight variants address thermal insulation and reduced dead load, while conductive or magnetic phase additions open routes to electromagnetic-interference shielding and self-sensing structural health monitoring; printable one-part formulations extend the same chemistry to additive manufacturing [7]. Our co-occurrence analysis, however, shows these functional streams to be comparatively under-developed—fibre-reinforcement and thermal resistance lead, while genuinely defence-specific functions (blast/ballistic resistance, EM shielding) appear in only a few dozen papers each—and, critically, none of them has yet been coupled to data-driven design. This is the white space the present framework targets.

## 3. Machine Learning for Geopolymer Property Prediction: State of the Art

Data-driven modelling has become the fastest-growing methodology in geopolymer research. The bibliometric survey identifies compressive strength prediction as the overwhelming focus, with artificial neural networks (ANNs) the most-used algorithm [9,10,21,22], followed by a rapidly rising second wave of tree ensembles (random forest, gradient boosting/XGBoost) and support vector machines [23,24]; gene expression programming persists as a route to closed-form design equations [25,26]. Three limitations recur across this literature and motivate the design choices in Section 4. First, models are trained overwhelmingly on fly-ash/slag two-part chemistries [11,27], bounding their transferability to one-part systems and secondary precursors; ML applied specifically to one-part and stabilized soil systems remains a recent, smaller literature [13,28,29,30]. Second, prediction targets are dominated by 28-day compressive strength, with fresh-state, durability, and functional properties comparatively neglected. Third, until recently, most models were reported as black boxes; the emergence of SHAP and related interpretability methods is the key development that turns a predictive model into a design tool by quantifying how each mixture variable pushes performance up or down [12,14]. The demonstration that follows is constructed to embody the maturing best practice—multi-algorithm comparison, cross-validated evaluation, and SHAP-based interpretation—while applying it explicitly to the one-part design space.

## 4. Machine Learning Demonstration

### 4.1. Dataset

We perform a new comparative and interpretable ML analysis of a published one-part geopolymer dataset—we contribute the modelling, evaluation, and interpretation, not new experimental data. The dataset comprises 80 fly-ash/GGBS one-part geopolymer paste mixtures compiled from twelve open-literature sources by Faridmehr et al. [31], all activated with a solid (anhydrous sodium metasilicate) alkaline source requiring only the addition of water—that is, one-part chemistry throughout, not the two-part liquid activator systems that dominate most ML studies. This places the demonstration within the small but active one-part × ML frontier [32,33,34,35,36,37,38,39,40]. Each record reports the 28-day compressive strength with five mixture and processing descriptors: fly-ash content (%), GGBS content (%), Na_2_O dosage of the solid activator (% by binder weight), water/binder ratio, and curing temperature. Unlike a single-laboratory factorial, the composition axis spans the full practical range (fly-ash 0–100%, GGBS 0–100%, Na_2_O 1.5–12.5%, w/b 0.20–0.50, curing 20–60 °C), and measured strengths cover 2.0–102.1 MPa (mean 45.9 MPa)—a genuine cross-study range with no missing values. The trade-off is size: 80 mixtures is modest for ML, so we evaluate conservatively and report cross-study generalization rather than over-reading in-sample fit (below).

### 4.2. Models and Evaluation

To avoid unstable attribution to two collinear precursor terms (Section 4.4), the composition axis was encoded as a single slag fraction, GGBS/(fly-ash + GGBS)—that is, the “slag” in this descriptor is the GGBS content of the mix, consistent with the terminology fixed in Section 1, giving four modelling descriptors: slag fraction, Na_2_O dosage, water/binder ratio, and curing temperature. Collapsing the pair to a ratio discards the total precursor mass, which varies only in the eight mixes whose precursors do not sum to 100% (range 80–100%, standard deviation 3.6 percentage points across the dataset); as a sensitivity check, we refit the headline model with total precursor mass fraction restored as a fifth descriptor and obtained an essentially unchanged LOSO R^2^ of 0.609 (versus 0.612), confirming that no material predictive information is lost by the ratio encoding in these data. The 80 mixtures were split 80/20 into training (*n* = 64) and held-out test (*n* = 16) sets. Four regression algorithms spanning the complexity spectrum were trained: ordinary linear regression (a transparent baseline), a support vector regressor (SVR) with an RBF kernel, a random forest, and gradient-boosted trees (XGBoost). Continuous features were standardized within a scikit-learn pipeline for the linear and kernel models.

Hyperparameters for the headline comparison were fixed a priori, not tuned, to keep the comparison transparent: SVR (C=100, ε=0.5, RBF kernel, γ = scale); random forest (500 trees, default depth); XGBoost (400 trees, learning rate 0.05, max depth 3, subsample 0.85). A single random seed (42) governs the train/test split and all stochastic model components. Software versions: Python 3.11, scikit-learn 1.9, XGBoost 3.2, SHAP 0.51, NumPy 2.4.

Because fixed settings could in principle favour one model over another, we additionally performed a nested cross-validation: within every LOSO fold, each model’s hyperparameters were re-tuned by an inner grouped three-fold grid search over the training studies only (SVR: C∈{1,10,100}, ε∈{0.1,0.5,1.0}; random forest: 100–300 trees, depth ∈{3,6,unlimited}; XGBoost: 50–300 trees, depth ∈{2,3}, learning rate ∈{0.05,0.1}), and the tuned model predicted the held-out study. The nested-CV LOSO results (XGBoost R^2^ = 0.60, SVR 0.56, random forest 0.55, linear 0.36; Table S3) reproduce both the ranking and the magnitudes obtained with fixed settings, indicating that the fixed hyperparameters do not confer an unfair advantage on any model. We also probed model capacity directly: sweeping XGBoost from 10 to 800 trees at depths 2 and 3, LOSO R^2^ rises to a plateau of 0.59–0.62 by roughly 50–100 trees and neither improves nor degrades thereafter (Table S3), and an early-stopped variant (validation-based stopping, median 59 trees) scores LOSO R^2^ = 0.56. Nominal parameter counts overstate the effective capacity of a shallow, shrunk (η=0.05), subsampled ensemble, and the flat capacity curve shows that the random split-to-LOSO gap is not an overfitting artefact of model size—it persists unchanged at one-tenth the capacity—but reflects between-study distribution shift, which we quantify in Section 4.3.

Because the dataset pools twelve independent source studies, we evaluated generalization at two levels: a conventional random 80/20 split with five-fold cross-validation, and a stricter leave-one-source-out cross-validation (LOSO), in which every mix from one source study is held out in turn and predicted from a model trained only on the other eleven. LOSO is the honest test of transfer to a new laboratory and mix design—it prevents near-replicate mixes from the same study leaking between train and test, and it is genuinely harder than a random split. We report both, with LOSO as the headline. The five recorded descriptors resolve the 80 records into 53 unique descriptor vectors: 38 records fall into 11 groups that share an identical five-descriptor vector with at least one other record, and identical-descriptor records map to strengths differing by up to 34.5 MPa. These are not duplicated measurements but distinct mixes (or replicate determinations) that five coarse descriptors cannot distinguish—unrecorded factors such as precursor fineness and chemistry, activator grade, and testing protocol differ between them. Two consequences follow. First, the within-group strength spread furnishes an empirical replicate noise floor: the pooled within-group standard deviation is ≈9.0 MPa, so no model on these five descriptors can be expected to achieve a cross-study RMSE much below ≈9 MPa (the observed LOSO RMSE is 15.5 MPa, of which this irreducible component is ≈34% of the error variance). Second, the effective number of independent design points is 53 (59 if records are kept separate across source studies), not 80, and we treat it as such: as a de-duplication sensitivity analysis, we averaged the strengths of identical-descriptor records within each source study (*n* = 59) and re-ran the full LOSO evaluation, obtaining XGBoost R^2^ = 0.59 (RMSE 15.3 MPa) versus 0.61 on the full data, with the model ranking unchanged—the headline conclusions do not rest on the duplicated vectors. Because LOSO holds out whole source studies, none of these near-duplicates leak across LOSO folds; they can, however, inflate the random split score, a further reason to foreground LOSO.

### 4.3. Results

Table 1 reports performance at three evaluation levels. On the random split, the ensemble models led—XGBoost reached test R^2^ = 0.90 (RMSE = 8.3 MPa) and the random forest R^2^ = 0.84, ahead of the linear baseline (0.80). Under the stricter leave-one-source-out test, which measures transfer to a study never seen in training, XGBoost remained the best model at LOSO R^2^ = 0.61 (RMSE = 15.5 MPa), ahead of the random forest (0.58), the SVR (0.54), and linear regression (0.36). To convey the precision of the headline figure, we report 95% bootstrap confidence intervals on the pooled LOSO predictions (4000 resamples): resampling records, R^2^ = 0.61 [0.44, 0.75] and RMSE = 15.5 [12.1, 18.6] MPa; resampling whole source studies (a cluster bootstrap, the more conservative choice for grouped data), R^2^ [0.38, 0.79] and RMSE [9.9, 20.2] MPa. The intervals are wide, as expected at *n* = 80 from twelve heterogeneous sources, and readers should weight the RMSE—which is robust to the variance of the held-out pool—at least as heavily as R^2^. The value of non-linear modelling is clearest at this level: the ensemble models hold a ≈0.22–0.25 R^2^ margin over linear regression under LOSO, whereas the random split gap is small (0.90 vs. 0.80)—so it is the transfer test, not the in-sample fit, that shows the strength response is materially non-linear in a way that generalizes.

**Figure 4:**
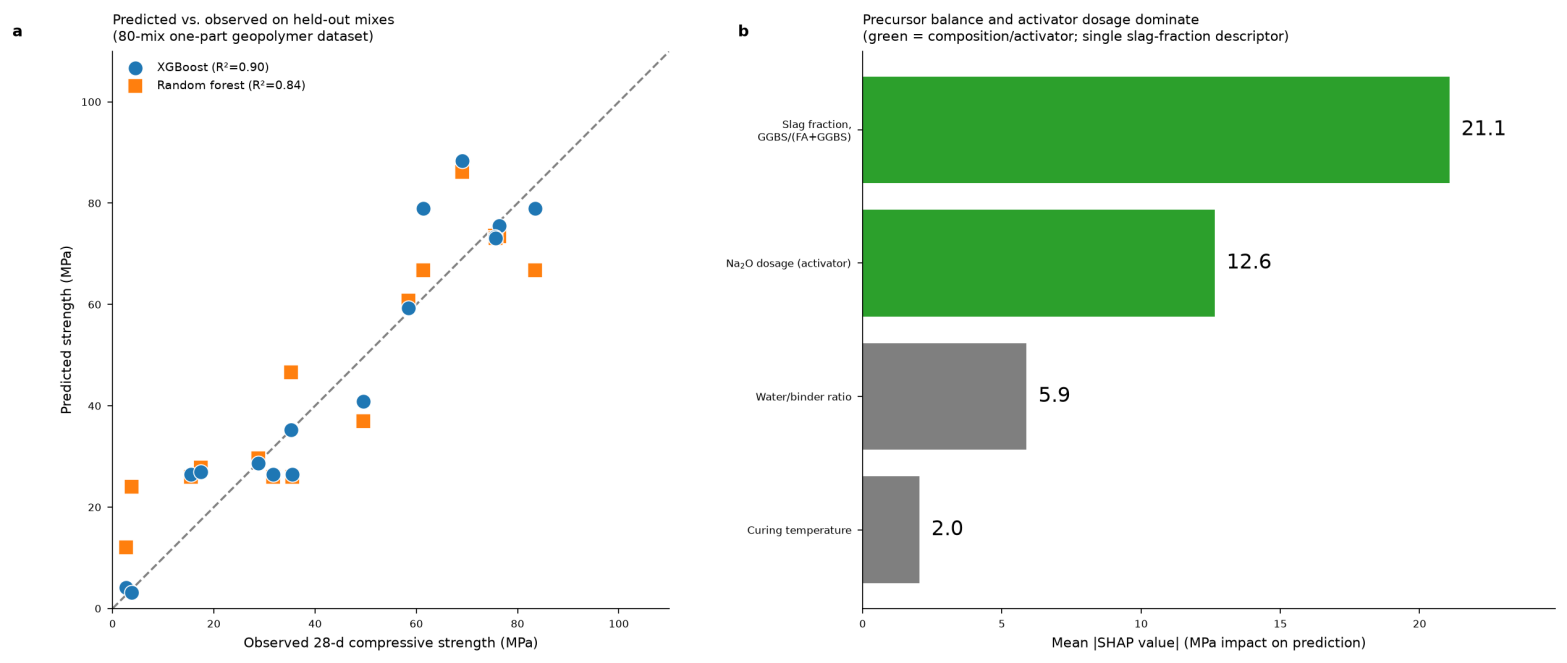
ML demonstration on the 80-mix one-part geopolymer dataset. (**a**) Predicted vs. observed 28-day compressive strength for the two leading models on held-out data (*n* = 16); dashed line is parity. (**b**) SHAP mean absolute feature importance from the XGBoost model, computed on the re-engineered feature set in which the collinear fly-ash/GGBS pair is collapsed to a single slag fraction. The precursor balance and Na_2_O activator dosage (green = composition/activator) dominate the prediction.

We lead with the LOSO and cross-validated figures rather than the single held-out test R^2^: with n=80 and only 16 test mixes, the test-set value is sensitive to the particular split (one or two points move it appreciably), and the grouped and cross-validated estimates are the more reliable measures of generalization. The fold-by-fold LOSO scores are themselves highly variable—individual held-out studies (*n* = 2–14 mixes each) yield fold R^2^ ranging from strongly negative to 0.99 (Table S2)—because R^2^ on a handful of held-out points is unstable; the pooled LOSO R^2^ over all 80 predictions and the five-fold mean are the meaningful aggregates, and per-fold RMSE (1–26 MPa) is a steadier per-study read than per-fold R^2^. The drop from random split to LOSO quantifies how much of the in-sample fit reflects similarity between mixes from the same laboratory. The LOSO error itself mixes physically distinct sources, which we separate by a variance decomposition of the pooled LOSO residuals (Table S4): the systematic per-study offset (the mean signed error of each held-out study, capturing laboratory bias and any formulation regime the training pool lacks) accounts for 23% of the LOSO mean-squared error, while the remaining 77% is within-study scatter—of which the replicate noise floor identified above (≈9 MPa among identical-descriptor mixes) alone accounts for roughly a third of the total error variance. The headline LOSO R^2^ = 0.61 should therefore be read as a lower bound on the transferable physical signal recoverable from five descriptors: part of what the model fails to predict is not predictable from these descriptors at all, and part is systematic study-to-study bias rather than failed physics. Conversely, we do not claim that 0.61 measures pure physical generalization, and we temper all cross-study claims accordingly. Figure 4a shows predicted-versus-observed strength for the two leading models about the parity line across the full 2–102 MPa range; fold-by-fold LOSO results and source-study group sizes (*n* = 2 to 14) are tabulated in Table S2 of the Supplementary Material.

### 4.4. Interpretation via SHAP

A feature engineering choice precedes the interpretation. In this dataset, the fly-ash and GGBS contents are near-perfectly anti-correlated (Pearson r=−0.99; 72 of 80 mixtures have fly-ash + GGBS =100%, with a secondary component filling the balance in the remaining eight), so they carry a single compositional degree of freedom, not two. Attributing importance separately to “fly-ash content” versus “GGBS content” under such collinearity is unstable and not independently meaningful. We therefore collapse the precursor axis to one descriptor, the slag fraction GGBS/(fly-ash + GGBS), and compute SHAP on that re-engineered four-feature model; the collinearity is removed by construction rather than merely flagged.

TreeSHAP applied to the re-engineered XGBoost model quantifies each descriptor’s mean absolute impact on the predicted strength (Figure 4b). Composition and activator terms dominate: the slag fraction is the single largest contributor (mean |SHAP| = 21.1 MPa), followed by Na_2_O activator dosage (12.6 MPa), then water/binder ratio (5.9 MPa) and curing temperature (2.0 MPa). We stress what this analysis is and is not. SHAP values are attributions of a fitted model’s predictions; they identify the dominant statistical predictors of strength in this dataset, not established causal effects. The observed ordering is consistent with known activation chemistry—the fly-ash/slag balance sets the calcium and aluminosilicate supply that governs gel formation, and the Na_2_O dosage controls the alkalinity that drives dissolution and condensation—and it agrees with the sensitivity analysis reported by the dataset’s original authors [31]. That an interpretable model recovers, from held-out data, a predictor ranking that independent chemistry would predict is encouraging, and it generates concrete, testable design hypotheses: that the precursor balance and the solid activator’s Na_2_O dosage are the highest-leverage variables for tuning a just-add-water mix. But observational, literature-pooled data cannot exclude confounding (for example, studies that used high slag fractions may also have used finer precursors or different testing conditions), so these hypotheses remain to be confirmed by controlled experiments; the active learning stage of the framework (Section 5, stage 5) is precisely the step that converts such correlational rankings into validated causal design knowledge, and we label it as such. The narrow tier occupied by curing temperature reflects the dataset’s emphasis on ambient and near-ambient curing, the regime most relevant to field deployment. Extending this same analysis to multi-property targets (durability, thermal resistance, shielding) is exactly the wide-range curation that Section 5 prescribes.

## 5. An AI-Assisted Design Framework for Multifunctional One-Part Geopolymers

**Figure 5:**
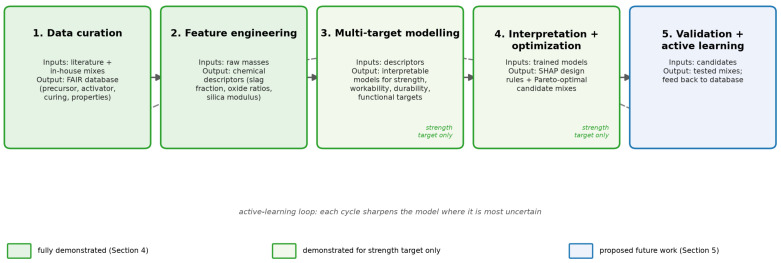
The five-stage AI-assisted design framework, with inputs and outputs at each stage and an active learning feedback loop. Shading distinguishes what this work demonstrates from what it proposes: stages 1–2 are demonstrated in full; stages 3–4 are demonstrated for the compressive strength target only (badged); stage 5 and the multi-target/optimization extensions are proposed future work.

The demonstration generalizes into a closed-loop framework with five stages (Figure 5). Before describing them, we state plainly what is *demonstrated* versus what is *proposed*, since the two must not be conflated. Section 4 demonstrates stages 1–2 in full (a curated 80-mix dataset and chemically motivated feature engineering, including the slag fraction descriptor) and demonstrates stages 3–4 for the single target of compressive strength (one interpretable model plus SHAP-based interpretation). It does not demonstrate multi-target modelling, formal multi-objective optimization, or the experimental active learning loop; those are the framework’s proposed extensions and remain future work. Figure 5 marks this boundary explicitly. With that scope fixed: **(1) Data curation:** assemble mixture descriptors and multi-property targets from the literature and in-house experiments into a standardized, FAIR-compliant database, explicitly recording precursor source, activator chemistry, curing, and—crucially—functional properties beyond compressive strength. **(2) Feature engineering:** encode composition as chemically meaningful descriptors (oxide ratios, reactive-phase fractions, silica modulus) rather than raw masses, improving transferability across precursor sources. **(3) Multi-target modelling:** train interpretable models not only for strength but for workability, setting time, durability, and functional targets (ductility, thermal resistance, shielding effectiveness), embracing the multi-objective nature of real design—early examples already predict freeze–thaw resistance and fibre-reinforced ductility of one-part systems [41,42,43]. **(4) Interpretation and optimization:** use SHAP and partial-dependence analysis to extract design rules [14], then apply multi-objective optimization (e.g., Bayesian optimization or genetic algorithms over the trained surrogates) to propose candidate formulations on the performance Pareto front. **(5) Experimental validation and active learning:** synthesize and test the highest-value candidates, feed results back into the database, and iterate—each cycle sharpening the model where it is most uncertain. This loop converts one-part geopolymer development from serial trial-and-error into a guided search, and because it is built on interpretable models, it yields transferable design knowledge, not just point predictions.

So that no claim outruns its evidence, Table 2 maps every substantive claim made in the title, abstract, and framework onto its supporting evidence and its status in this work.

## 6. Limitations

The strength of the conclusions is bounded by the demonstration’s scope, which we state plainly. **(i) Dataset size and effective sample size.** Eighty mixtures is small for machine learning, and the five descriptors resolve them into only 53 unique design points (Section 4.2), which is the honest measure of the information available; individual LOSO folds are correspondingly noisy, and the models should be read as an illustrative benchmark, not a production predictor. **(ii) Literature-derived heterogeneity and source bias.** The data pool twelve studies with differing raw materials, mixing protocols, and testing practices; source-study effects are real—which is precisely why we evaluate by leave-one-source-out and decompose the LOSO error into between-study bias (23%) and within-study scatter (77%; Section 4.3)—but they also limit how far any single trained model transfers. **(iii) Feature collinearity.** The fly-ash and GGBS contents are near-perfectly anti-correlated (r=−0.99); we mitigate this by modelling a single slag fraction descriptor, but the precursor axis remains a single degree of freedom in these data and cannot be resolved into independent fly-ash and slag effects, and in the eight mixes whose precursors do not sum to 100%, the ratio encoding cannot recover the absolute contents (though restoring the total precursor mass as a feature leaves the results unchanged; Section 4.2). **(iv) Strength-only target.** Only 28-day compressive strength is modelled; the durability, ductility, thermal, shielding, and blast/ballistic properties invoked by “multifunctional” and “defence”framing are reviewed and built into the proposed framework but are not modelled here for want of comparable open data. **(v) Paste-level data.** The mixtures are geopolymer pastes, not concretes; aggregate, scale, and structural effects are not represented. **(vi) No experimental validation.** No new specimens were synthesized or tested; the active learning loop of Section 5 is proposed, not executed. **(vii) Publication bias.** The dataset derives exclusively from published studies, and the literature preferentially reports successful, higher-strength formulations: weak mixes are under-represented (the low tail of the 2–102 MPa range is sparse), so the model is least constrained exactly where formulations fail, and the SHAP ranking may be tilted toward the levers that matter in the well-performing region of the design space. Predictions in the low-strength regime should therefore be treated with extra caution. Mitigations belong to the framework’s curation stage: deliberately including the grey literature, theses, and negative results when assembling future databases, and, where the truncation is severe, using censored/truncated regression formulations rather than ordinary least-squares-style objectives. These limitations motivate the framework rather than undercut it: each is a specific target for the data curation, multi-target modelling, and validation stages the framework prescribes.

## 7. Conclusions

One-part geopolymers are among the most field-deployable low-carbon binder classes available, well-suited to the defence and civil infrastructure settings where liquid activator handling is impractical. Their formulation space, however, is too large and too strongly interacting for trial-and-error development. This paper has reviewed the materials science of one-part geopolymer composites and, on a published 80-mix one-part geopolymer dataset, demonstrated that interpretable machine learning predicts 28-day compressive strength—and only that property—with useful accuracy: a held-out test R^2^ = 0.90 that, under the more reliable leave-one-source-out test of transfer to a new laboratory, settles to XGBoost R^2^ = 0.61 (95% bootstrap CI 0.44–0.75; RMSE 15.5 MPa), well above a linear baseline (0.36)—a ranking that survives nested hyperparameter tuning and de-duplication sensitivity analysis, and a gap from the random split score that capacity sweeps attribute to between-study heterogeneity rather than overfitting. After collapsing the collinear fly-ash/GGBS pair to a single slag fraction descriptor, SHAP recovers a predictor ranking consistent with activation chemistry (the precursor balance and the solid activator’s Na_2_O dosage as the dominant statistical predictors of strength in these data; their causal confirmation is assigned to the framework’s experimental validation stage). We stress the boundaries of this evidence: it is strength-only, at paste level, on 80 literature-pooled mixtures, and it does not yet substantiate the durability, ductility, thermal, shielding, or blast/ballistic performance that “multifunctional” and “defence”applications ultimately demand. Those targets are the subject of the proposed—not demonstrated—AI-assisted design framework (Section 5). The principal opportunity revealed by our bibliometric analysis is nonetheless clear: although ML for geopolymer strength prediction is now well-established, the great majority of that work addresses conventional two-part mixes and compressive strength alone, leaving the one-part and multifunctional design space comparatively underexplored. Extending interpretable, multi-target data-driven design toward field-deployable multifunctional one-part composites is a tractable and high-impact direction, and the framework and worked strength example here are offered as a concrete first step toward it.

## Figures and Tables

**Table 1:** Predictive performance of the four regression models on the 80-mix one-part geopolymer dataset, using the re-engineered feature set (precursor axis collapsed to a single slag fraction). The random split column measures in-sample interpolation; five-fold CV and leave-one-source-out (LOSO) measure generalization, with LOSO the honest transfer test to an unseen source study. Best LOSO values are for XGBoost. All models used fixed (untuned) hyperparameters and random seed 42.

Model	Test R^2^	RMSE (MPa)	MAE (MPa)	5-Fold CV R^2^ (±SD)	LOSO R^2^
XGBoost	0.90	8.3	5.9	0.65 ± 0.13	0.61
Random forest	0.84	10.5	8.7	0.60 ± 0.21	0.58
Support vector regression	0.71	14.2	9.7	0.62 ± 0.09	0.54
Linear regression	0.80	11.9	10.5	0.41 ± 0.23	0.36

**Table 2:** Claim-by-claim evidence map. “Demonstrated” means supported by the analyses of Section 4 of this paper; “reviewed” means supported by the cited literature; “proposed” means put forward as a framework element or future direction without new empirical support here.

Claim	Evidence	Status
Interpretable ML predicts 28-day compressive strength of one-part fly-ash/slag pastes with useful cross-study accuracy	LOSO R^2^ = 0.61 [95% CI 0.44–0.75], RMSE 15.5 MPa on 80 published mixes (Table 1)	Demonstrated
Non-linear models transfer across studies better than a linear baseline	LOSO margin of ≈0.22–0.25 R^2^ over linear regression; confirmed under nested tuning (Table S3)	Demonstrated
Slag fraction and Na_2_O dosage are the dominant strength predictors	SHAP ranking (Figure 4b), concordant with [31] and activation chemistry	Demonstrated as statistical association; causal status is a hypothesis pending stage-5 validation
Framework stages 1–2 (curation, feature engineering)	Executed on the 80-mix dataset (Section 4)	Demonstrated
Framework stages 3–4 (modelling, interpretation/optimization)	Executed for the single strength target; no multi-target or formal optimization	Demonstrated for strength only
Framework stage 5 (experimental validation, active learning)	None in this work	Proposed
Multifunctional/defence-relevant performance (ductility, thermal, shielding, blast)	Literature review (Section 2.3); no new data or models here	Reviewed and proposed as target

## Data Availability

The original contributions presented in this study are included in the article/Supplementary Material. Further inquiries can be directed to the corresponding author.

## Supplementary Materials

The following supporting information can be downloaded at: https://www.mdpi.com/article/10.3390/ma19153347/s1.
