# Supplementary material for: Interpretable Machine Learning for One-Part Fly-Ash/Slag Geopolymer Strength Prediction: Toward Multifunctional Binder Design
Source: Materials (Basel). 2026 Aug 6;19(15):3347. doi: 10.3390/ma19153347 (PMC13467817; doi:10.3390/ma19153347)
Supplement: Supplementary file 1 [file materials-19-03347-s001.zip › materials-4452968-supplementary.pdf]

Supplementary Materials

Interpretable Machine Learning for One-Part Fly-Ash/Slag Geopolymer Strength Prediction: Toward Multifunctional Binder Design

Vinoth Nageshwaran, Sudhir Amritphale, and Soundararajan Ezekiel

This document provides the supplementary tables referenced in the main text. Tables S3 and S4 were added during revision in response to peer review.

Table S1: Bibliometric screening and tagging summary (PRISMA-style). Records were retrieved from the Crossref REST API via eight bibliographic queries, pooled, and de-duplicated by DOI. Relevance and sub-stream tagging used case-insensitive regular expressions applied to record *titles* (the corpus retains title-level metadata; abstracts were not stored). The 1,850 on-topic papers are the subset of the 4,312 de-duplicated records whose titles match the on-topic regex; no other screening step intervenes.

Screening / tagging stage	Count
Records retrieved (8 Crossref queries, pooled)	≈6,400 (with duplicates)
Unique records after DOI de-duplication	4,312
On-topic (geopolymer / alkali-activated regex), 2014–mid-2026	1,850
— tagged ML stream	346
— tagged one-part stream	308
— one-part AND ML	17
Records with title text >120 characters (concept network)	1,092
Validation sample (title-based adjudication)	100

Regex-tag validation on the 100-record adjudication sample gave precision = 1.00 for both the ML and one-part streams (no false positives), with recall = 0.76 (ML) and 0.86 (one-part); the residual errors are recall misses on records whose titles omit the trigger terms. Because tagging is title-based, this validation is conservative on recall.

Table S2: Leave-one-source-out (LOSO) fold-by-fold results for the XGBoost model on the 80-mix one-part fly- ash/GGBS geopolymer dataset. Each row holds out all mixes from one source study and predicts them from a model trained on the other eleven. Fold R^2 on a handful of held-out points is unstable and ranges from strongly negative to near-unity; per-fold RMSE is a steadier per-study read. The pooled LOSO R^2 over all 80 predictions is 0.61 (five-fold CV mean R^2 = 0.65).

Source study (ref.)	Mixes held out (n)	LOSO R^2	LOSO RMSE (MPa)
17	6	0.097	14.35
18	6	−0.154	12.45
19	4	−2.196	21.80
20	6	−2.720	9.33
21	4	0.673	6.94
22	2	−2.584	6.06
23	3	0.996	1.01
24	6	0.474	13.07
25	14	−0.349	9.30
26	11	0.852	6.13
27	7	0.060	25.26
28	11	−0.218	25.61
**Total / pooled**	**80**	**0.612**	**15.49**

Source-study reference numbers correspond to the citation keys in the main-text reference list. The wide spread of per-fold R^2 (from −2.72 to 0.996) reflects the small size of individual held-out groups (n = 2 to 14); the pooled LOSO R^2 and the five-fold cross-validated mean are the meaningful aggregate measures of generalization.

Table S3: Hyperparameter-tuning and model-capacity analyses (added in revision). *Top:* nested cross-validation— within every LOSO fold, hyperparameters are re-tuned by an inner grouped 3-fold grid search over the training studies only, and the tuned model predicts the held-out study; the ranking and magnitudes reproduce the fixed- hyperparameter comparison of Table 1. *Bottom:* pooled LOSO performance of XGBoost across a capacity sweep (learning rate 0.05, subsample 0.85). Performance plateaus by ≈50–100 trees and neither improves nor degrades up to 800 trees, so the random-split-to-LOSO gap is not an artefact of model size. An early-stopped variant (20% validation split within each fold, patience 30; median 59 trees) gives pooled LOSO R^2 = 0.56, RMSE = 16.6 MPa.

(a) Nested cross-validation (tuned within each LOSO fold)

Model	LOSO R^2	LOSO RMSE (MPa)
XGBoost	0.595	15.83
SVR (RBF)	0.556	16.57
Random forest	0.549	16.71
Linear regression	0.355	19.97

(b) XGBoost capacity sweep (pooled LOSO R^2 / RMSE, MPa)

Trees	Max depth 2		Max depth 3	
	R^2	RMSE	R^2	RMSE
10	0.309	20.68	0.331	20.35
25	0.530	17.06	0.558	16.55
50	0.589	15.95	0.610	15.54
100	0.597	15.79	0.619	15.36
200	0.602	15.69	0.613	15.48
400	0.613	15.48	0.612	15.49
800	0.607	15.60	0.611	15.51

Table S4: Variance decomposition of the pooled LOSO error of the headline XGBoost model (added in revision). For each held-out study the signed mean error (bias) captures systematic study-level offset; the residual within-study scatter is what remains after removing that offset. Weighted by group size, between-study bias accounts for 23% of the pooled LOSO mean-squared error and within-study scatter for 77%; the replicate noise floor (≈9.0 MPa pooled within-group SD among the 11 groups of identical-descriptor mixes, Section 4.2 of the main text) alone corresponds to ≈34% of the total error variance and is irreducible for any model restricted to these five descriptors.

Source study (ref.)	*n*	Bias (MPa)	Within-study RMSE (MPa)
17	6	9.45	10.80
18	6	−6.28	10.75
19	4	18.07	12.19
20	6	−4.69	8.07
21	4	−2.98	6.26
22	2	−6.06	0.13
23	3	−0.30	0.97
24	6	−9.18	9.30
25	14	8.43	3.94
26	11	0.84	6.07
27	7	−8.10	23.92
28	11	−3.14	25.42
**Decomposition of pooled LOSO MSE (239.8 MPa^2):** between-study bias 23%, within-study 77%			
**Replicate noise floor:** ≈81 MPa^2 (≈34% of pooled MSE)			
